# Immunogold labeling of synaptic vesicle proteins in developing hippocampal neurons

**DOI:** 10.1186/s13041-020-0549-x

**Published:** 2020-01-20

**Authors:** Jung-Hwa Tao-Cheng

**Affiliations:** NINDS Electron Microscopy Facility, National Institute of Neurological Disorders and Stroke, National Institutes of Health, Bethesda, MD 20892 USA

**Keywords:** Electron microscopy, Axon transport, Synaptophysin, SV2, Synapsin, Active zone cytomatrix

## Abstract

Synaptic vesicles (SV) contain high concentrations of specific proteins. How these proteins are transported from soma to synapses, and how they become concentrated at SV clusters at presynaptic terminals were examined by immunogold electron microscopy in dissociated rat hippocampal neurons at 3–6 days in culture, a developmental stage when axonal transport of SV proteins is robust. In neuronal somas, labels for the SV integral membrane proteins (synaptophysin, SV2, VAMP/synaptobrevin, and synaptotagmin) were localized at Golgi complexes and other membranous structures that were dispersed in the cytoplasm as individual vesicle/vacuoles. These vesicles/vacuoles became aggregated in axons, with the size of aggregates ranging from 0.2 to 2 μm in length. Pleomorphic vesicle/vacuoles within the aggregate were typically larger (50–300 nm) than SVs, which were uniform in size at ~ 40 nm. These pleomorphic vesicles/vacuoles are probably transport cargos carrying SV integral membrane proteins from the soma, and then are preferentially sorted into axons at early developmental stages. Serial thin sections of young axons indicated that many labeled aggregates were not synaptic, and in fact, some of these axons were without dendritic contacts. In contrast, labels for two SV-associated proteins, synapsin I and α-synuclein, were not localized at the Golgi complexes or associated with membranous structures in the soma, but were dispersed in the cytoplasm. However, these SV-associated proteins became highly concentrated on clusters of SV-like vesicles in axons, and such clusters were already distinctive in axons as early as 3 days in culture. These clusters consisted of ~ 4–30 vesicles in single thin sections, and the vesicles were of a uniform size (~ 40 nm). Serial sectioning analysis showed that these clusters could be part of nascent synapses or exist in axons without any dendritic contact. Importantly, the vesicles were intensely labeled for SV integral membrane proteins as well as SV-associated proteins. Thus, these EM observations reveal that the two groups of proteins, SV integral membrane and SV-associated, proceed through different routes of biosynthesis and axon transport, and are only sorted into the same final compartment, SV clusters, when they are in the axons.

## Introduction

The transport of presynaptic proteins from soma through axon to their final destination at presynaptic terminals is complex and a subject of intense study [1, 2]. The presynaptic specializations consist of clusters of synaptic vesicles (SV) and active zone (AZ) cytomatrix, which are localized at sites of SV release. While the transport of AZ proteins has been described at both the light microscopy (LM) and electron microscopy (EM) levels [3–5], few EM studies have been carried out on SV proteins, especially in developing axons. Although a few immunogold EM images of some SV proteins were shown to demonstrate their presence in the AZ transport aggregate [5], that study did not include detailed ultrastructural description on these SV proteins’ biosynthesis or axonal transport.

Fluorescence tagged SV proteins like GFP-synaptophysin [6] and GFP-VAMP/synaptobrevin [7] provided live observations of packets of tubular/vesicular structures carrying these SV proteins and moving through axons. Correlative LM immunolabeling of these mobile packets showed the presence of other presynaptic proteins indicating that the packets contain many components required for the formation of the presynaptic terminal [7]. Correlative EM of these mobile packets showed aggregates of tubular-vesicular structures, pleomorphic small vesicles, and dense core vesicles (DCV) [7]. However, direct visualization of SV proteins on the various components via immunogold labeling by EM is lacking.

In axons of cultured rat neurons younger than 3 days in vitro (DIV), GFP-tagged SV proteins have a diffused appearance with few stationary puncta, which represent nascent synapses [7]. From 4 DIV onward, many mobile transport packets move up and down the axons, and the number and size of nascent synapses increase with time [7]. The present study used pre-embedding immunogold EM to examine the distribution of various endogenous SV integral membrane proteins including synaptophysin [8], SV2 [9], VAMP/synaptobrevin [10], synaptotagmin [11]; and SV-associated proteins including synapsin [12] and synuclein [13] in dissociated rat hippocampal neurons. Young axons at 3–6 DIV were chosen for easier identification of mobile transport packets, which outnumber synapses at these developmental stages [7].

The present approach of examining the distribution of endogenous SV proteins provides a clear view of these proteins’ biogenesis and transport at the ultrastructural level. These observations illustrate the different routes taken by different SV and AZ proteins, and provide clues to their eventual incorporation into a nascent synapse.

## Methods

### Antibodies

Rabbit polyclonal antibody (rabbit pAb) against synaptophysin (1:250) was from DAKO (Glostrup, Denmark); mouse monoclonal antibody (mouse mAb) against SV2 (1:500) was a gift from Dr. Erik S. Schweitzer (UCLA, Los Angeles, CA); mouse mAb against VAMP (1:100, clone SP10) and SNAP-25 (1:250, clone SP14) were from Chemicon (Temecula, CA); mouse mAb against synaptotagmin (p65, 1:250, clone ASV30) and mouse mAb against Bassoon (1:100, clone SAP7F407) were from Stressgen (Victoria, BC, Canada); mouse mAb against α-synuclein (1:100, clone 42) was from BD Biosciences (San Jose, CA); mouse mAb against synapsin I (1:250, clone 46.1) was from Synaptic Systems (Gottingen, Germany). Guinea pig polyclonal antibody against Piccolo (1:100) was a gift from Dr. Eckart Gundelfinger (Leibniz Institute for Neurobiology, Magdeburg, Germany).

### Preparation of rat dissociated hippocampal neuronal cultures

Most samples were from a previously published report on synaptic active zone cytomatrix proteins [5] and reexamined here for synaptic vesicle proteins. Briefly, cell cultures were prepared from embryonic 20-day-old rat fetuses by papain dissociation, and then plated with or without a glial feeder cultures, and experiments were carried out with 3–6 day-old cultures. No difference in labeling pattern was observed between the two types of cultures for any of the antibodies.

### Fixation, pre-embedding immunocytochemistry and electron microscopy

For optimal structural preservation, cells were fixed with 4% glutaraldehyde in 0.1 M cacodylate buffer at pH 7.4 for 30 min at room temperature and then stored at 4 °C. These samples were post-fixed with 1% osmium tetroxide in 0.1 M cacodylate buffer for 1 h on ice, and stained with 1% uranyl acetate in acetate buffer at pH 5.0 overnight before dehydration and embedding for electron microscopy.

For immunogold labeling, cells were fixed with one of the following fixation conditions (optimal fixation conditions for each antibody are listed in Additional file 1): (1) 4% paraformaldehyde in phosphate buffered saline (PBS) for 45–60 min, (2) 4% paraformaldehyde and 0.02–0.05% glutaraldehyde for 30–60 min, (3) 2% acrolein in PBS for 1 min followed by 4% paraformaldehyde in PBS for 30–60 min. Immunolabeling steps were carried out as described before [14] with some modifications on treatment time and concentrations of reagents. Briefly, fixed cells were washed and permeabilized/blocked with 0.1% saponin/5% normal goat serum in PBS for 1 h, incubated with primary antibody for 1–2 h, incubated with secondary antibody conjugated to 1.4 nm gold particles (1:250, Nanogold from Nanoprobes, Yaphand, NY) for 1 h, silver enhanced (HQ silver enhancement kit, Nanoprobes). Controls for specificity of immunolabeling include omitting the primary antibody and using the different primary antibodies as controls for each other.

Samples were then treated with 0.2% OsO_4_ in phosphate buffer for 30 min on ice, followed by 0.25% uranyl acetate in acetate buffer at pH 5.0 at 4 °C for 30 min-1 h or overnight, dehydrated in a graded series of ethanol and embedded in epoxy resin. Thin sections were cut at ~ 70 nm thickness. Serial sections were collected on single-slot, film-coated grids based on methods detailed in Harris et al., 2006 [15]. Sections were counterstained with uranyl acetate and lead citrate. Images were photographed with a bottom-mounted digital CCD camera (AMT XR-100, Danvers, MA, USA) on a JEOL 1200 EX electron microscope.

### Identification criteria of neuronal soma, axon and dendrite

In dissociated hippocampal cultures that contain a mixture of neurons and glia, it is more difficult to distinguish the two cell types at 3–6 DIV than in older cultures. For example, the conspicuous structural differences of the nuclei between neurons and glia at 3 wk. in culture [16] is not evident in the young cultures used in the present study. Thus, the identification of neuron vs. glia was based on antibody labeling in the present study, where all antibody used are neuron-specific. Furthermore, because all antibodies used here are specific for presynaptic proteins, the presence of label in neurites indicates that they are axons. This assumption is consistent with earlier reports of LM immunolabeling that synaptophysin and synapsin are sorted into axons early in development [17].

### Measurement of labeling density

Labeling densities were measured for the Golgi complex and cytoplasm of neuronal somas, and for clusters of SV-like vesicles in axons. Every neuronal soma encountered was photographed at 10,000x magnification, and every cluster of SV-like vesicles was photographed at 40,000x magnification. Density of label was calculated by counting all particles of label on the identified structure divided by the area, and expressed as number of particles per μm^2^. Area was measured with ImageJ (National Institutes of Health, Bethesda, MD, USA).

## Results

### Labels for SV integral membrane proteins are localized at the Golgi complex in the soma and sorted into vesicular structures in axon

Dissociated hippocampal neuronal cultures at 3–6 DIV were fixed and labeled with antibodies against four different SV integral membrane proteins: synaptophysin, SV2, VAMP & synaptotagmin. Synaptophysin and SV2 antibodies produced the most consistent and high efficiency labeling under many different fixation conditions, and thus, were illustrated to a greater extent in the present study.

**Fig. 1:**
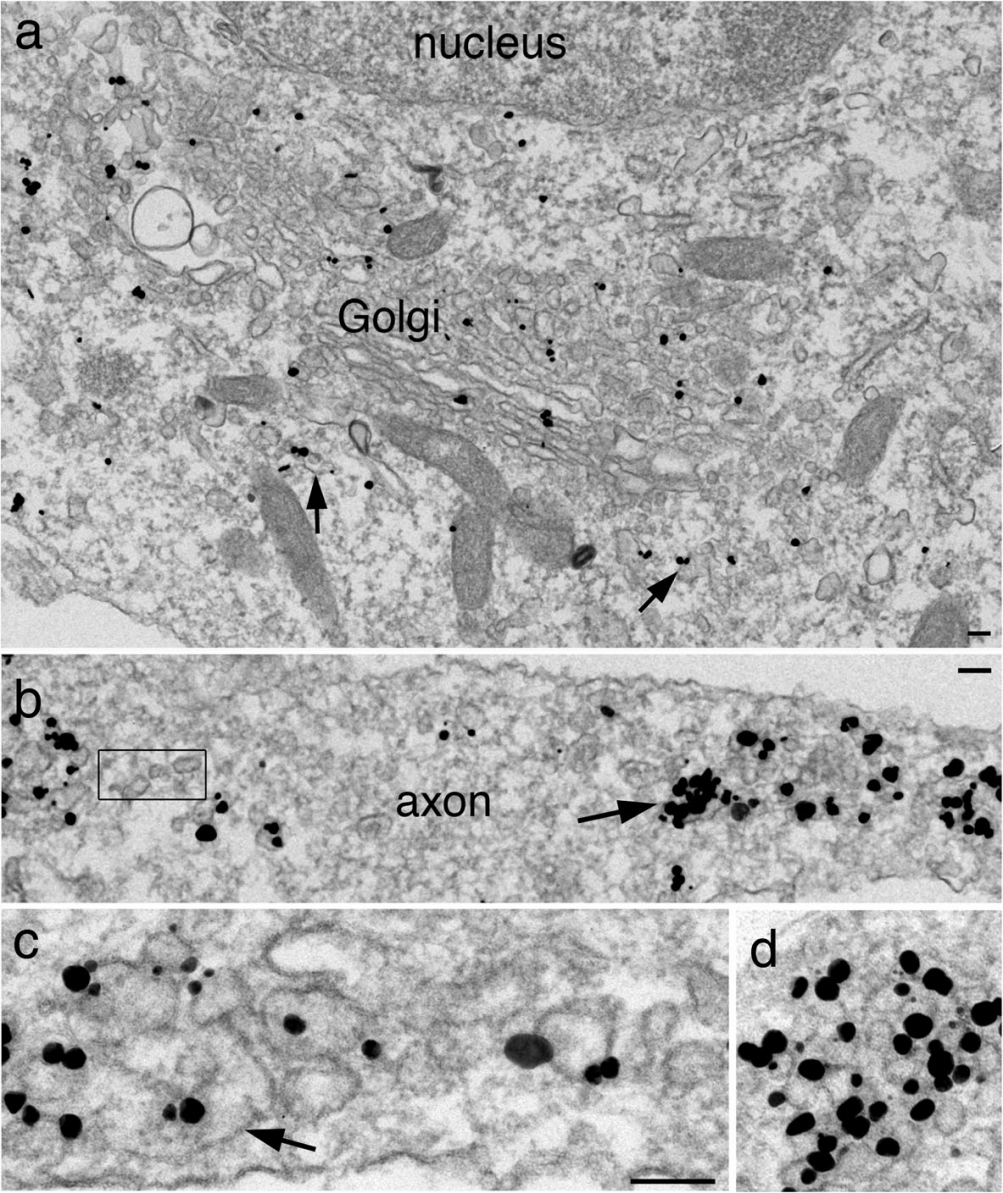
Immunogold labeling of synaptophysin in dissociated hippocampal neuronal culture at 4 (**a**, **b**) and 5 (**c**, **d**) DIV. In neuronal soma (**a**), label is localized at the Golgi complex and on membranous structures (arrows in a). In axons (**b**-**d**), label is concentrated on aggregates of vesicles/vacuoles (arrow in **b**). However, not all vesicles/vacuoles are labeled (boxed area in **b**). The labeled aggregates consist of tubular (arrow in c) and vesicular structures. Clusters of SV-like vesicles of uniform size (~ 40 nm) are also intensely labeled (**d**). Scale bars = 100 nm, **c** & **d** shared the same scale bar

**Fig. 2:**
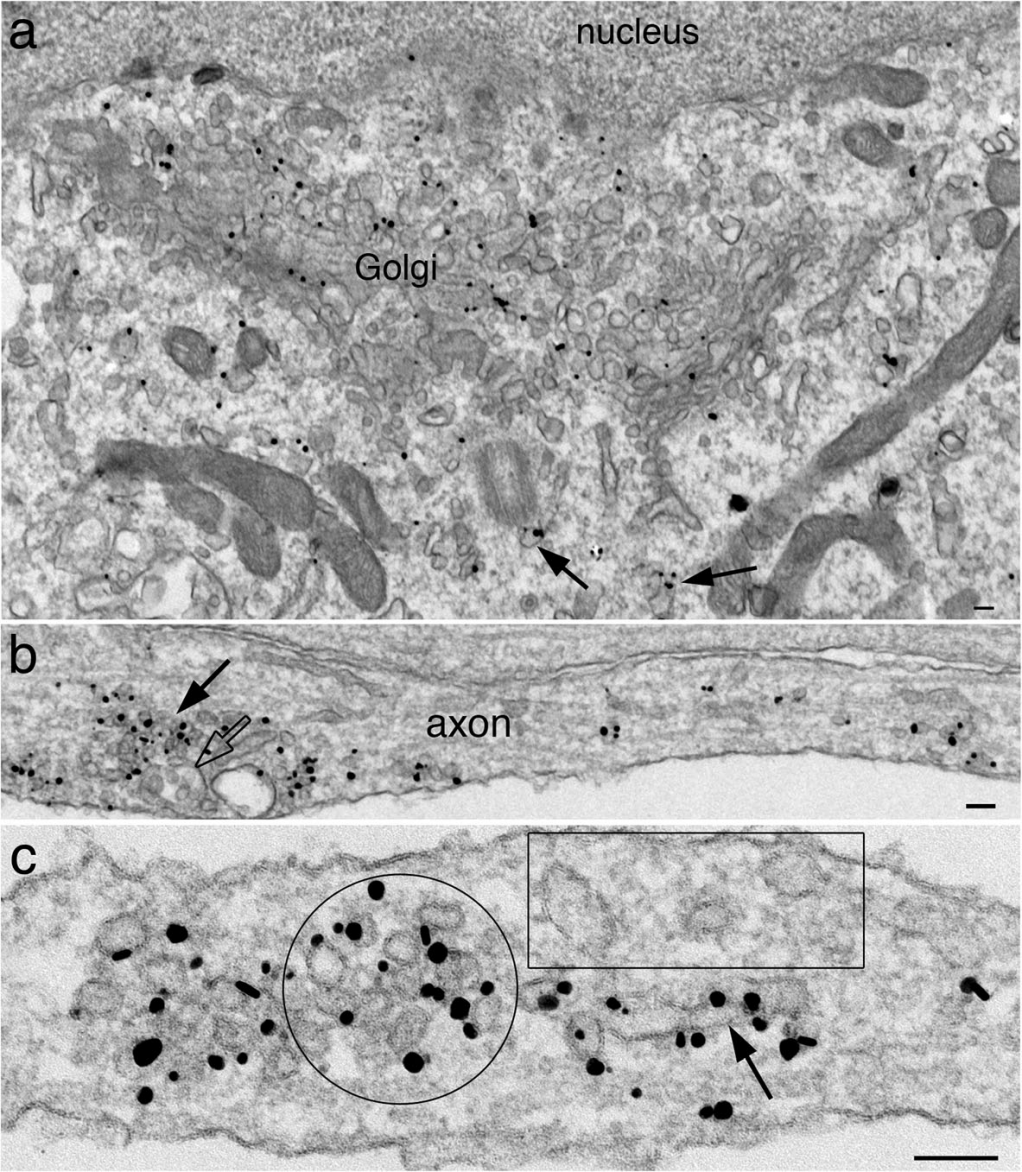
Label for SV2 is localized at the Golgi complex and membranous structures (arrows in **a**) in soma, and at individual and aggreges (arrow in **b**) of vesicles /vacuoles in axons. Enlarged micrograph in **c** shows concentrated label on a cluster of SV-like vesicle (circled area) and on a nearby tubular structure (arrow). Boxed area shows other nearby tubular-vesicular structures without any label. Samples were 4 DIV. Scale bars = 100 nm

As expected of integral membrane proteins, labels for synaptophysin (Fig. 1a) and SV2 (Fig. 2a) were localized at the Golgi complex [1]. Density of label was consistently 3–4 times higher at the Golgi complex than at nearby cytoplasm (Table 1). In neuronal somas, labels for both antibodies were also specifically localized at membranous structures of various size and shape, scattered in the cytoplasm as individual entities (arrows in Figs. 1a and 2a).

**Table 1:** Labeling densities (number of particles per μm^2^) of different SV proteins at the Golgi complex vs. cytoplasm in neuronal soma, and at clusters of SV-like vesicles in axon

	antibody		soma			axon
			Golgi	cytoplasm	Ratio G/C	clusters of SV-like vesicles
SV membrane proteins	synaptophysin	Exp 1	16.4 ± 1.4 (6)	4.5 ± 0.5 (8)	3.64**	395.5 ± 26.6 (13)
		Exp 2	11.1 ± 1.3 (6)	3.0 ± 0.4 (8)	3.70*	301.3 ± 25.1 (12)
	SV2	Exp 1	27.6 ± 3 .6 (5)	6.5 ± 0.8 (7)	4.25*	262.6 ± 30.9 (10)
		Exp 2	16.9 ± 1.7 (5)	4.7 ± 0.5 (5)	3.60*	411.6 ± 35.2 (11)
SV-associated proteins	Synapsin I	Exp 1	1.7 ± 0.1 (10)	4.8 ± 0.6 (12)	0.35**	158.4 ± 24.6 (8)
		Exp 2	3.4 ± 0.3 (5)	8.6 ± 0.9 (8)	0.40**	244.1 ± 27.0 (11)
	α-synuclein	Exp 1	4.0 ± 0.6 (5)	10.0 ± 1.0 (5)	0.40*	115.4 ± 13.2 (7)
		Exp 2	2.2 ± 0.4 (3)	6.7 ± 2.6 (3)	0.33 NS	181.3 ± 25.1 (6)

(n) = number of Golgi, neuronal somal cytoplasmic area, and clusters of SV-like vesicles sampled

Ratio G/C – labeling density of Golgi divided by that of cytoplasm. Difference in labeling density tested by Student t-test: * *P* < 0.005, **P < 0.0005, NS – not significant

Many of these labeled vesicles/vacuoles became aggregated in the axons (arrows in Figs. 1b and 2b), but not in soma and dendrites. These labeled aggregates are termed “SV membrane protein transport aggregate” in this paper. The overall size of the labeled aggregates ranged widely. Many comprised several vesicles/vacuoles (~ 0.2 μm, arrows in Additional file 2a & b), but many others exceeded 1 μm in length (arrows in Figs. 1b and 2b), and sometimes greater than 2 μm (Additional file 3a). Higher mag images of these labeled aggregates showed a mixture of tubular (arrows in Figs. 1c and 2c) and vesicular structures of variable size and shape.

**Fig. 3:**
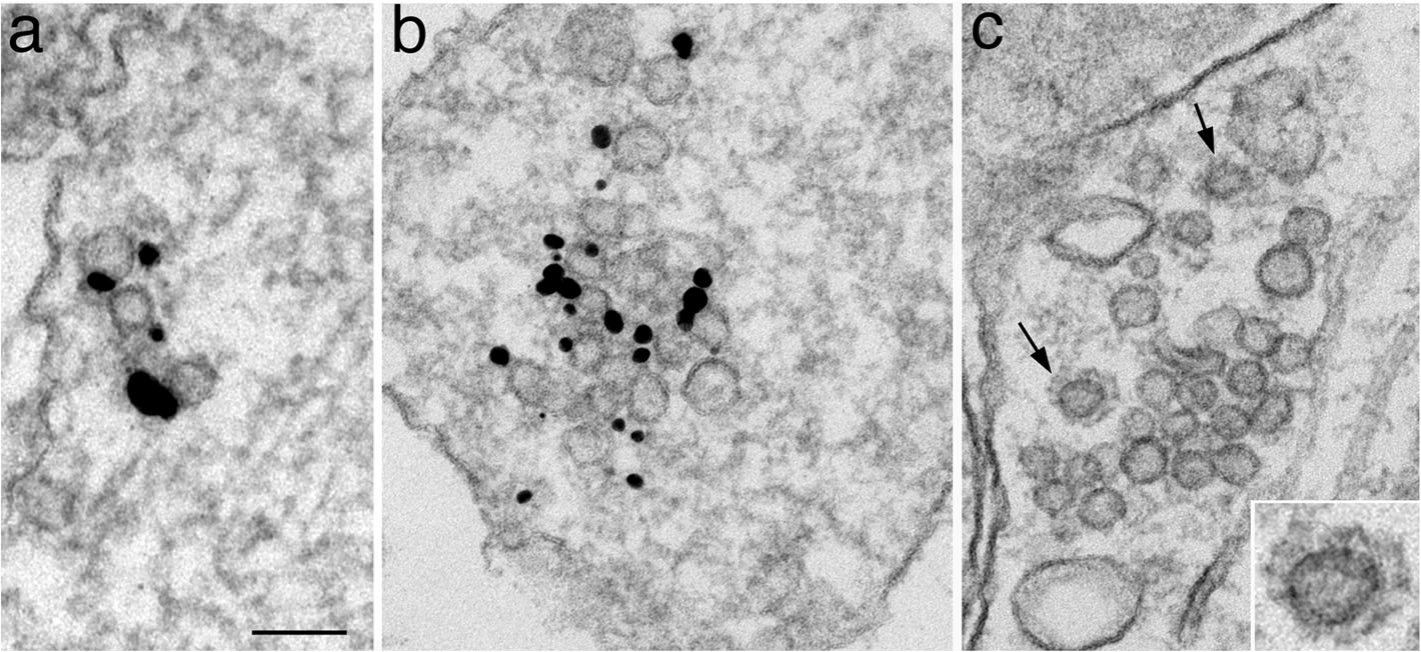
Clusters of SV-like vesicles are labeled for synaptophysin (**a**) and SV2 (**b**). Samples were 4 DIV, and (**c**) was fixed with glutaraldehyde for better structural preservation. Arrows in (**c**) points to coated vesicles interpreted as clathrin (enlarged in inset). Scale bar = 100 nm

Notably, clusters of vesicles of uniform diameter of ~ 40 nm were also labeled in axons (Fig. 1d; circled area in Fig. 2c) at very high labeling densities (Table 1). These vesicle clusters resemble synaptic vesicles (SV) clusters in the presynaptic terminals [18], and are termed “clusters of SV-like vesicles” here. The number of vesicles in these clusters ranged from 4 to 30 in single sections; examples from small and larger clusters are illustrated in Fig. 3. Interestingly, clathrin-coated vesicles were often present near these SV-like vesicle clusters (arrows in Fig. 3c), suggesting endocytoses [19].

**Fig. 4:**
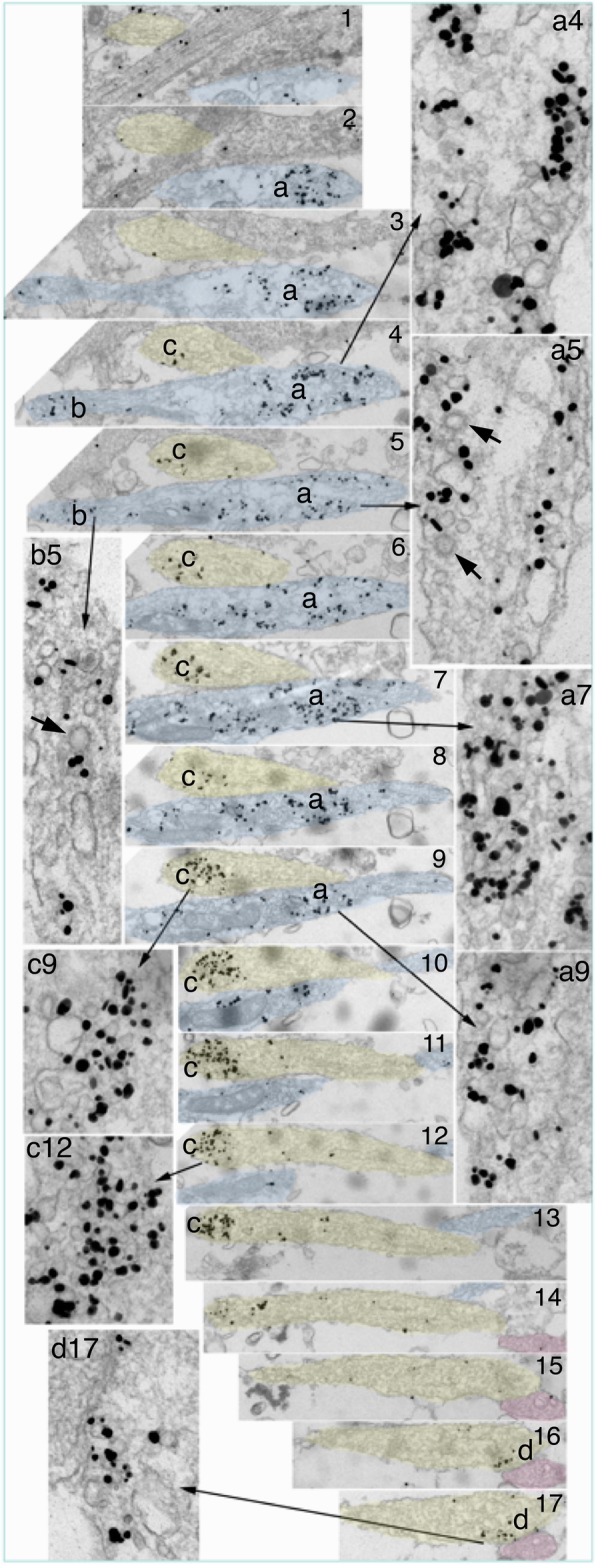
Serial sections (1–17 in center column) through two axons (yellow and blue) containing synaptophysin-labeled aggregates (marked as a, b & c) of vesicles/vacuoles at 5 DIV. Insets on both sides of the serial sections show enlargements of these aggregates at various sections. The axon segments containing aggregates a & c do not have contact with dendrites, whereas aggregate d in sections 16–17 is in contact with a dendrite (pink), perhaps forming a nascent synapse. Thick arrows in insets a5 & b5 point to clathrin-coated vesicles

Serial thin sections demonstrated that some labeled aggregates were clearly not part of synapses, and many axons containing these labeled aggregates did not even come in contact with dendrites (Fig. 4; Additional file 4). Thus, the aggregation of these labeled vesicles/vacuoles was intrinsic to the axon and not induced by external contact with dendritic elements. Some of these aggregates consisted mostly of tubular vesicular structures (aggregate “a” in Fig. 4), and others mostly of SV-like vesicles (aggregate “c” in Fig. 4). Interestingly, clathrin-coated vesicles were often seen among both types of labeled aggregates (thick arrows in Fig. 4), indicating active endocytosis near both types. Due to crowding of the vesicles/vacuoles, it is difficult to discern whether clathrin-coated vesicles were specifically labeled, though some coated vesicles appeared to be labeled (arrow in Additional file 4, section #4).

Multivesicular bodies (MVB, open arrows in Fig. 2b; Additional file 2c; Additional file 4, section # 4 & 5), which are considered late endosomes en route to fuse with lysosome [20], were often present among the labeled aggregates of vesicles/vacuoles. However, the great majority of these MVBs were not labeled for SV integral membrane proteins. Notably, no late stage lysosomes, such as lipofuscin bodies or vacuoles containing multilamellar structures or electron dense material [20], were observed in the vicinity of labeled SV protein transport aggregates.

Labels for two other SV integral membrane proteins, VAMP/synaptobrevin and synaptotagmin, were also localized at the Golgi complex (images not shown), and at individual and aggregated vesicles/vacuoles in the axons (Additional file 2c, d). Thus, the four SV integral membrane proteins studied here had similar distribution patterns in soma and in axon. However, the present preembedding immunogold labeling method does not allow double labeling, and thus, cannot determine whether these four proteins are colocalized in the same vesicle/vacuoles. Notably, not all vesicles/vacuoles were labeled even when they were in the vicinity of the labeled aggregates (boxed area in Figs. 1b, 2c and Additional file 2).

### Labels for SV-associated proteins are cytosolic in soma and become associated with clusters of SV-like vesicles in axon

**Fig. 5:**
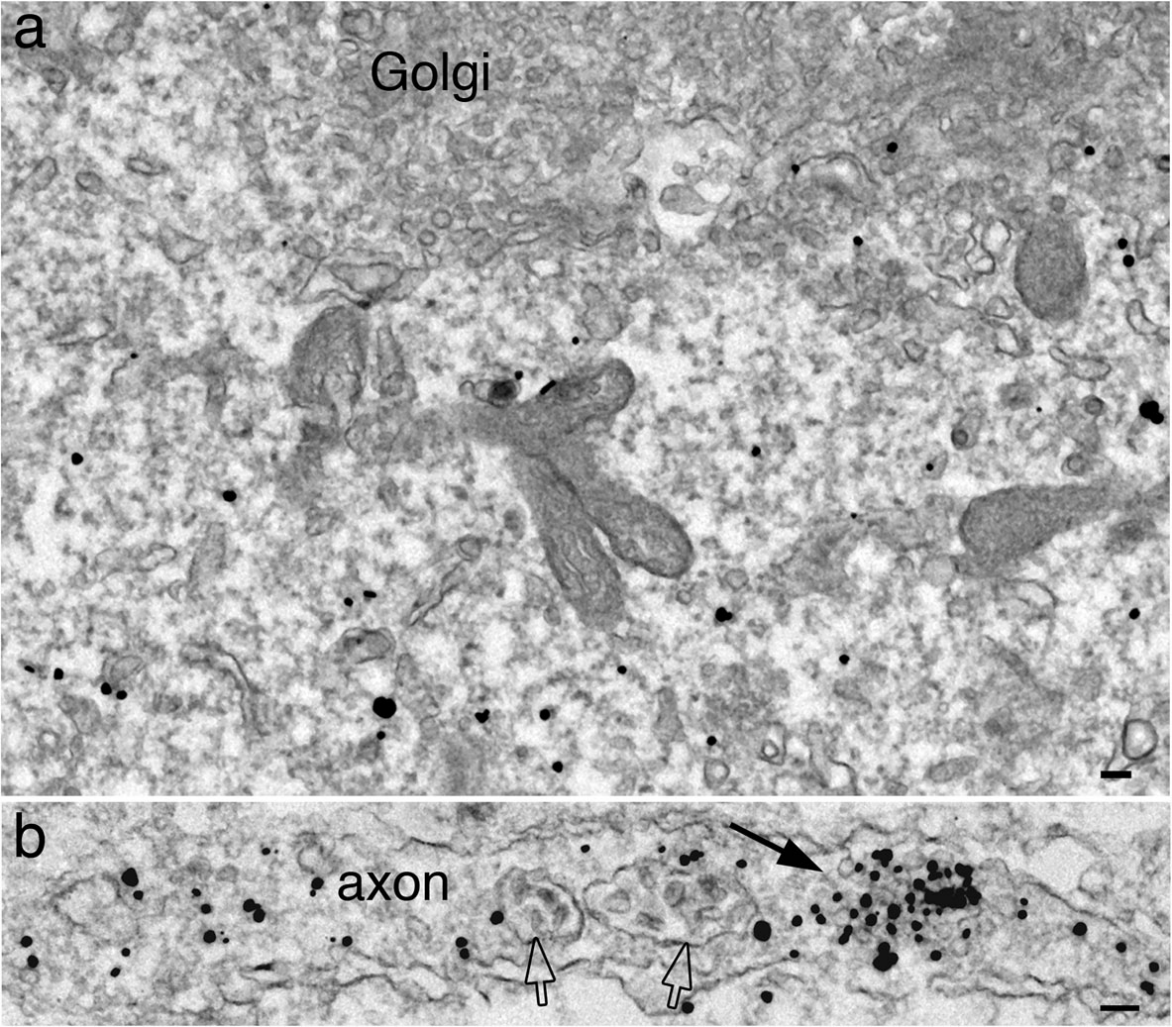
Label for synapsin I is not associated with the Golgi complex, but dispersed in the cytoplasm in the neuronal soma (**a**), and concentrated on clusters of SV-like vesicles (arrow in **b**) in axons. Open arrows point to multivesicular bodies (MVB) in **b**. Samples were 4 DIV. Scale bars = 100 nm

**Fig. 6:**
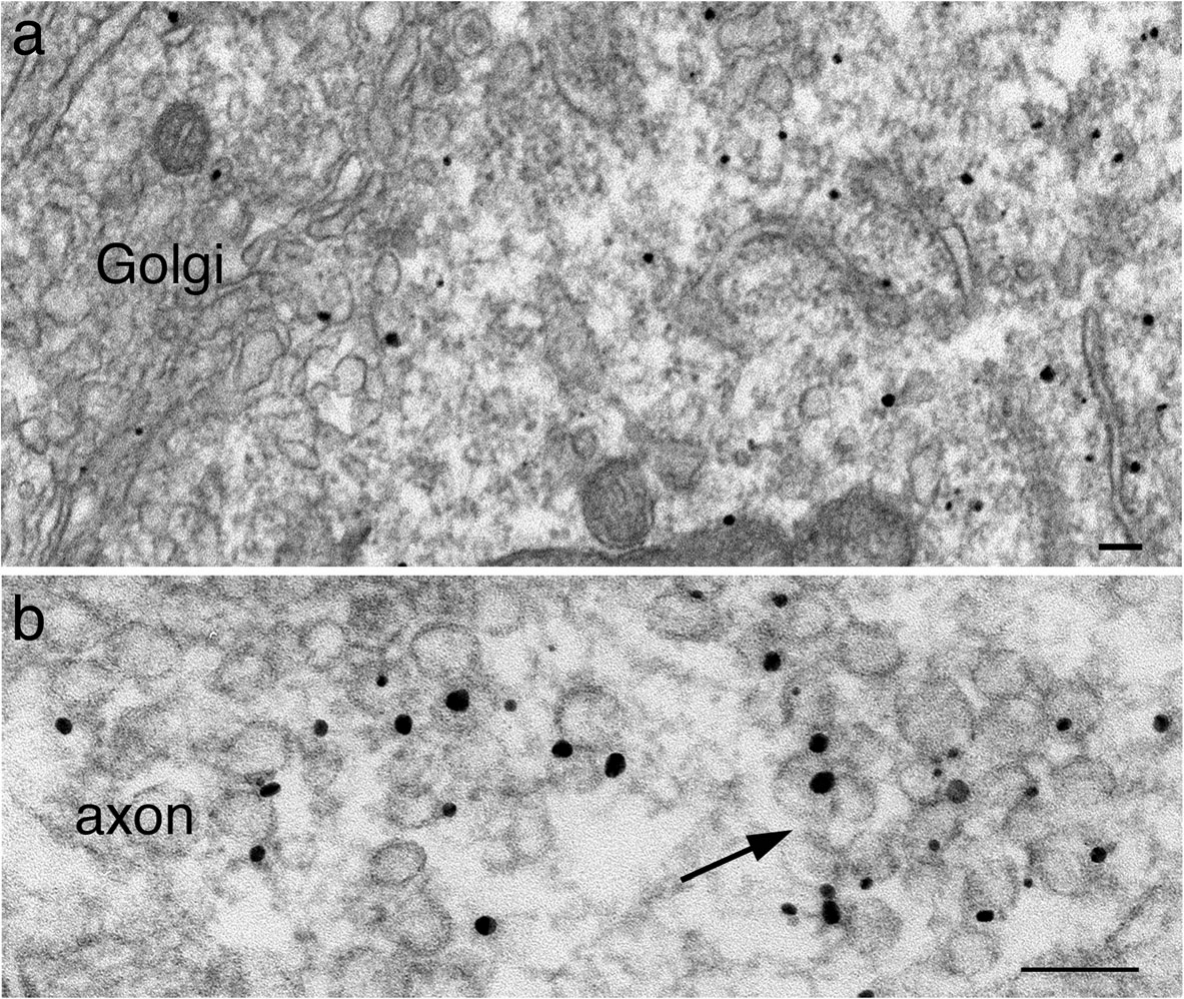
Label for α-synuclein is not associated with the Golgi complex, but dispersed in the cytoplasm in the neuronal soma (**a**), and concentrated on clusters of SV-like vesicles (arrow in **b**) in axons. Samples were 6 DIV. Scale bars = 100 nm

In contrast to labels for SV integral membrane proteins, labels for two SV-associated proteins, synapsin I and α-synuclein, were not concentrated at the Golgi complex (Figs. 5a and 6a) in the soma, but dispersed in cytoplasm, not associated with any membranous structures. The density of label at the Golgi complex was ~ 33–40% of that of the cytoplasm (Table 1), and perhaps represent background noise of immunogold labeling. The synapsin I antibody used here produced better labeling efficiency than the α-synuclein antibody, and thus, generated more detailed observations here.

In mature synapses, labels for synapsin I and α-synuclein are localized to clusters of SVs in the presynaptic terminals [18]. Here in young neuronal cultures before robust synaptogenesis occurs, most labels for synapsin I and α -synuclein were cytosolic in somas and axons, but became concentrated on clusters of SV-like vesicles in axons (arrow in Figs. 5b and 6b; Table 1). However, no other membranous structures, such as tubular or pleomorphic vacuoles were conspicuously labeled for synapsin I in the axons. Thus, it appears that synapsin I only become intensely associate with clusters of SV-like vesicles, consistent with the notion that synapsin I plays a role in clustering the SV vesicles [12].

### Label for SNAP 25 is sorted to axolemma early in development

**Fig. 7:**
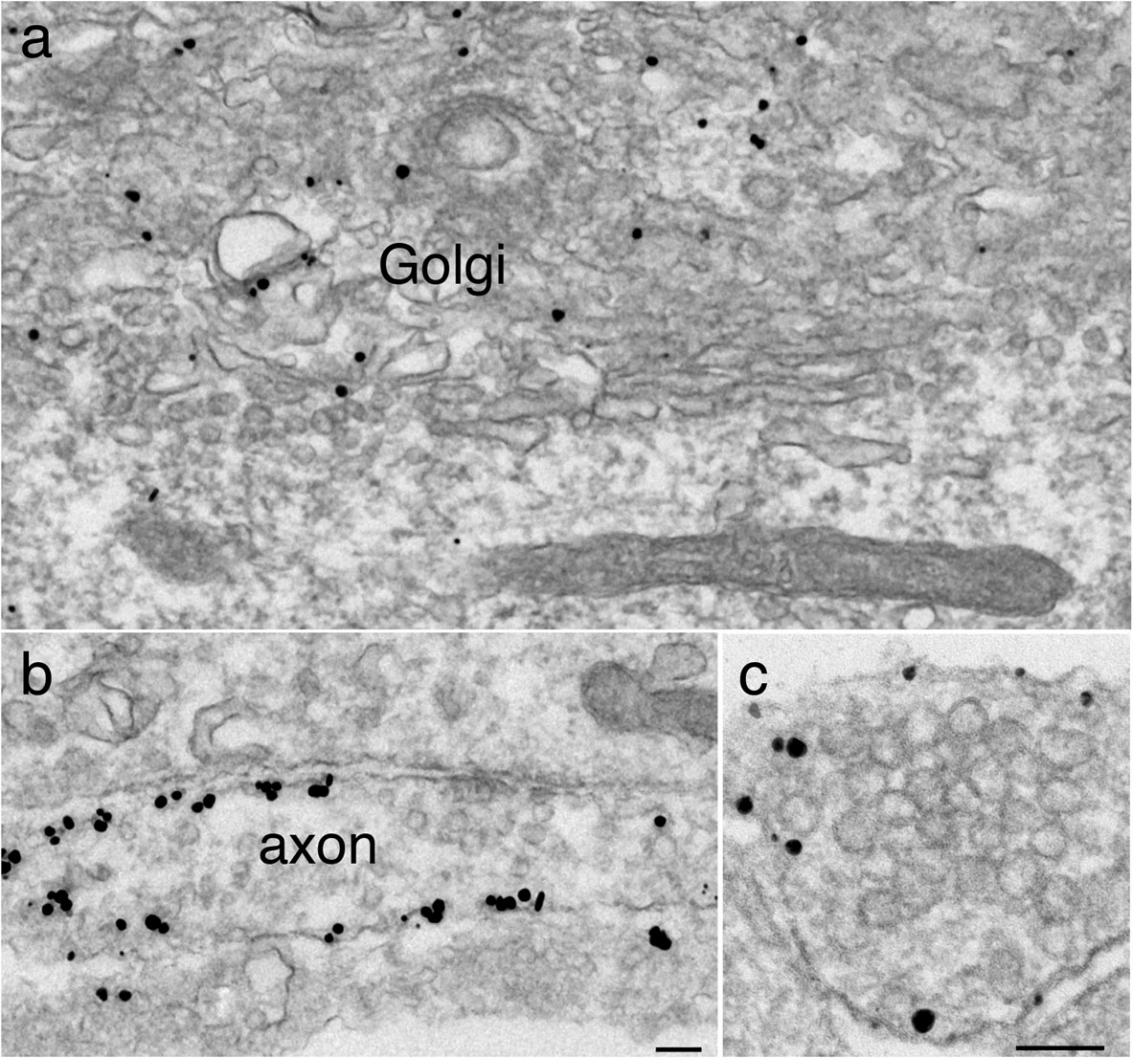
Label for SNAP-25 is localized at the Golgi complex in the soma at 4 DIV (**a**), and preferentially at axolemma (**b**, **c**), but not at clusters of SV-like vesicles (**c**) in axons. Scale bars = 100 nm, a & b share the same bar

SNAP-25, synaptosomal associated protein of 25 kDa, is part of the SNARE complex involved in exocytosis of synaptic vesicles [10]. In mature neurons, label for SNAP-25 is polarized to axon and localized to plasma membrane along the entire axon [21, 22]. Here, in young neuronal cultures, labeling pattern of SANP-25 was compared to those of the SV proteins illustrated above. Label for SNAP-25 was localized at the Golgi complex in soma (Fig. 7a), and became sorted to axolemma (Fig. 7b, c) as early as 4 DIV. In contrast to SV proteins, clusters of SV-like vesicles were clearly not labeled (Fig. 7c).

### Presynaptic terminals of immature synapses contain a full complement of SV and AZ proteins

**Fig. 8:**
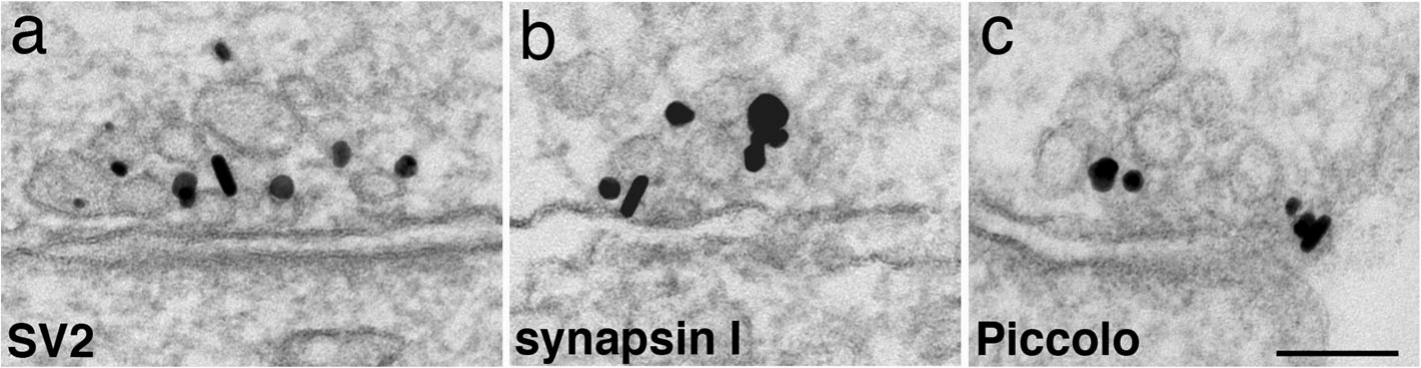
Nascent synapses are formed as early as 3 (**a**), 4 (**b**) and 5 (**c**) DIV, with a few synaptic vesicles and some larger sized vesicles/vacuoles. These vesicles label for SV proteins, SV2 (**a**), SV-associated protein, synapsin I (**b**), and AZ cytomatrix, Piccolo (**c**). Scale bar = 100 nm

Nascent synapses were seen as early as 3 DIV (Fig. 8a) and synaptic profiles appeared more frequently in subsequent days. The immature synapses contained fewer SVs than mature synapses [5, 23], but already showed a characteristic synaptic cleft with a uniform gap at ~ 20 nm, and a postsynaptic density of variable prominence (Fig. 8). As in the case of mature synapses [18], the SVs were labeled for both the SV integral membrane proteins (Fig. 8a) and SV-associated proteins (Fig. 8b). Thus, even though SV integral membrane proteins and SV-associated proteins were transported via different routes from soma through axons, they ended up in the same final compartment, the SVs, at the presynaptic terminal upon synapse formation. Furthermore, labels for AZ cytomatrix proteins also localized at active zone in these nascent synapses [5] (Fig. 8c).

### Dense core vesicles are frequent in developing axons, and contain some but not all SV proteins

**Fig. 9:**
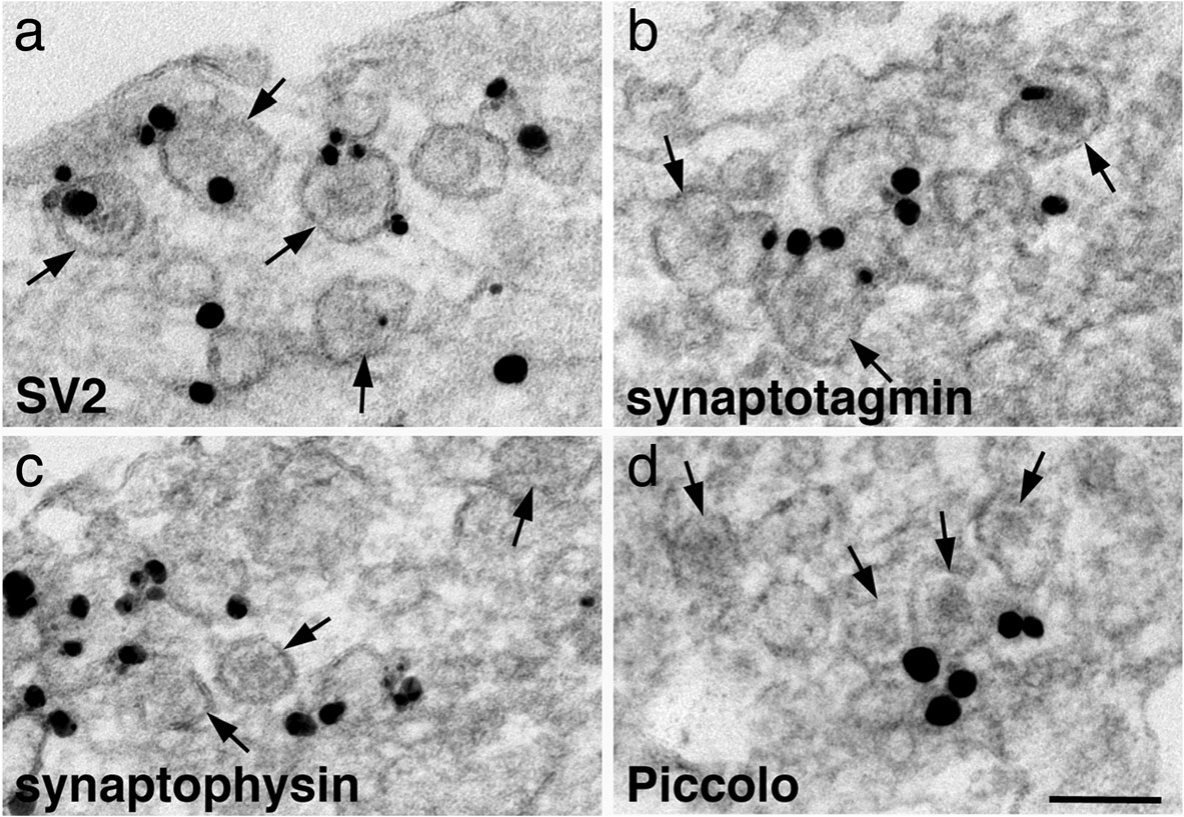
Dense core vesicles (DCV, arrows) sometimes exist in groups in young axons at 3 (**c**), 4 (**a**, **b**) and 6 (**d**) DIV. The vesicular membranes of DCV label for SV2 (**a**) and synaptotagmin (**b**), but not for synaptophysin (**c**). Label for Piccolo, an AZ cytomatrix protein is located on the outside of the DCV (**d**). Scale bar = 100 nm

Dense core vesicles (DCV) are more frequently seen in young axons than in mature samples, both in animals [23] and in cell cultures [3, 5, 7]. These DCVs sometimes existed in groups, intermingled with some SV-like vesicles (Fig. 9). Because occurrence of these vesicle mixtures of multiple DCVs and SVs were relatively infrequent compared to the occurrence frequencies of SV or AZ protein transport aggregates, it is difficult to capture them for serial section analysis. Thus, it cannot be determined whether these vesicle mixtures are part of a developing presynaptic specialization or existed in isolation in the absence of dendritic contact. While the SV-like vesicles labeled for all SV proteins, the vesicular membranes of DCV labeled for SV2 (Fig. 9a) and synaptotagmin (Fig. 9b), but not for all SV membrane proteins. For example, DCV were mostly negative for synaptophysin (Fig. 9c) or VAMP (image not shown), and labels for AZ cytomatrix proteins were localized to dark material outside of DCV (Fig. 9d) [5].

## Discussion

The present study used immunogold EM to examine distribution of endogenous synaptic vesicle (SV) proteins in young axons in dissociated hippocampal cultures at 3–6 DIV to document these proteins’ biosynthesis, axon transport, and eventual sorting into the SV.

The contrast between SV integral membrane proteins and SV-associated proteins was striking. In the neuronal soma where proteins are typically synthesized, labels for SV integral membrane proteins (synaptophysin, SV2, VAM/synaptobrevin, and synaptotagmin) were localized at the Golgi complex [24] and other membranous structures in the cytoplasm. These findings are consistent with the idea that membrane proteins are synthesized by ribosomes on the rough endoplasmic reticulum, trafficked through the Golgi complex, and sorted into vesicular cargos [25]. In contrast, labels for SV-associated proteins (synapsin and synuclein) were not localized at the Golgi [24], but were dispersed in the cytoplasm, not associated with membranous vesicles/vacuoles. These findings are consistent with the idea that cytosolic proteins are synthesized by free ribosomes in the cytoplasm. Here, these two different routes of protein syntheses were illustrated in developing neurons as early as 3 DIV at the ultrastructural level.

SV integral membrane and SV-associated proteins were both polarized into axons early in development [17], but the axonal transport of these two types of proteins is very different [1, 26]. SV integral membrane proteins are transported as a mixture of tubular-vesicular structures by fast axonal transport [6, 7], predominantly carried by Kinesin 3 family [1]. The present EM immunogold study corroborates previous axonal transport studies finding that label for SV membrane proteins are always associated with vesicular structures. However, not all SV membrane proteins are sorted into the same vesicular cargos, as synaptophysin and SV2 are transported separately in spinal nerve bundles [27] and in differentiated PC12 cells [28]. On the other hand, axonal transport for SV-associated proteins (synapsin and synuclein) is even more complicated [1, 26] because these proteins are reversibly associated with SV membranes. In general, cytosolic cargos are in the slow transport component and membrane-associated vesicular cargos are in the fast axonal transport component [26]. The present study provided ultrastructural evidence that label for synapsin I in axons was mostly cytosolic, and only became associated with vesicular membranes after clusters of SV-like vesicles were formed. These results are consistent with the observation that the bulk of synapsin moves in the slow component, and only 10–15% of synapsin moves in the fast component [26].

The present results suggest that SVs with a full complement of their specific proteins are not formed in the soma but only in the axon, and can form in the absence of dendritic contact. These observations are consistent with earlier reports that SVs are formed only after undergoing exo- and endocytosis through specialized sorting at recycling endosomes in axons, and can form without dendritic contact [1, 29, 30]. The common occurrence of clathrin-coated vesicles near the SV transport aggregates provides structural evidence for robust endocytosis at these locations. The fact that many clathrin-coated vesicles were of a similar size to the clusters of SV-like vesicles nearby is consistent with the possibility that these coated vesicles could shed the clathrin coating and become SV-like vesicles [19]. Furthermore, the axolemmal labeling of SNAP-25, a part of the SNARE complex involved in exocytosis [10], is consistent with the idea that exocytosis can occur all along the axon, not just restricted to presynaptic active zone [21]. Thus, the present study suggests that young axons could be capable of localized exocytosis and endocytosis, resulting in clusters of SV-like vesicles at non-synaptic sites.

In addition to SV proteins, the active zone (AZ) cytomatrix proteins, such as Bassoon and Piccolo, also have to be transported through axons to reach their final destination at the synapses [2–5]. These AZ transport aggregates consist of 1–2 dense core vesicles (DCV) and 4–5 SV-like vesicles in single sections. The average size of these AZ transport aggregates (~ 0.2 μm) [5] is much smaller than the SV membrane protein transport aggregates reported here, which often exceeded 1 μm in length (Additional file 3). Although LM immunolabeling showed partial colocalization of SV and AZ transport cargos [3, 31], these studies were not focused on the relative amount of SV vs. AZ proteins in transit. The present study demonstrated that the amount of label for SV proteins is much greater in the larger sized SV transport aggregates, and thus, the bulk of SV proteins are transported via the SV but not the AZ transport aggregates.

Notably, DCVs are consistently present in AZ transport aggregates, and AZ proteins like Bassoon and Piccolo are associated with the outside of the DCV membrane [5]. It has been proposed that a nascent presynaptic active zone can be formed by the exocytosis of a few DCV [4] or AZ transport aggregate [5]. It is likely that exocytosis of these DCVs would deposit the externally associated AZ material onto the cytosolic side of the plasma membrane, forming an AZ-like structure. Whether such AZ-like structures precede dendritic contact is still unresolved. If so, such “orphan” active zones would have Bassoon or Piccolo-labeled dark material localized to the cytoplasmic side of axonal plasma membrane without an apposed dendritic element. No such “orphan” AZ-like structures were seen in young axons 3–6 DIV by EM examination [5], but they could exist in cultures older than 10 DIV, where Bassoon-labeled “orphan” puncta are present by LM evidence [30]. Finally, many more DCVs are present in developing than in mature axons [3, 5, 7, 23], and multiple DCVs are sometimes seen at nascent presynaptic terminals [5] but rarely in mature ones [32]. The depletion of DCVs in mature axons suggests that DCVs are exocytosed during development, and could possibly play a role in synaptogenesis [32].

Interestingly, multivesicular body (MVB), a vacuole of the late endosome category [20], was frequently seen in close association with the SV protein transport aggregate. This observation is consistent with LM observations on axons from young hippocampal cultures that ~ 85% of anterogradely transported SV puncta colocalize with lysosome-related puncta [31]. The lysosome-related marker used in that study is Lamp1, which labels MVBs even before their fusion with lysosomes [33]. Thus, the MVB seen in the present study near SV membrane transport aggregates may represent the Lamp1-labeled “lysosome-related vesicles” [31]. In that study, loss of the lysosomal kinesin adaptor led to accumulation of SV and AZ proteins in the soma and a decrease of these proteins in the presynaptic sites, suggesting that a lysosome-related organelle may be involved in presynaptic biogenesis [31]. The present finding that these MVBs did not labeled for SV proteins suggests that SV proteins may not traffic through these MVBs.

In summary, the present findings provide ultrastructural data supporting the views that (1) SV integral membrane proteins (synaptophysin, SV2, VAMP, synaptotagmin) and SV-associated proteins (synapsin I and synuclein) are transported in axons via different routes, the former in aggregates of tubular-vesicular structures and the latter mostly cytosolic, (2) SV-associated proteins only become membrane-associated after SV-like vesicles with a uniform size at ~ 40 nm are formed, (3) clusters of SV-like vesicles are not formed in soma but in axon, (4) these clusters of SV-like vesicles contain a full complement of SV-specific proteins, and can form in young axons prior to dendritic contact. The present study also provides additional evidence that the SV transport aggregates are distinct from clusters of SV-like vesicles or AZ transport aggregates, and that the bulk of SV proteins are transported via SV transport aggregate.

## Supplementary information

**Additional file 1.** Optimal fixation conditions for each antibody.

**Additional file 2.** Immunogold labeling of SV proteins in young axons.

**Additional file 3.** Comparison among SV membrane protein transport aggregate, AZ protein transport aggregate, and cluster of SV-like vesicles.

**Additional file 4.** Serial sections through a young axon at 5 days in culture labeled with SV2 antibody.

## Data Availability

The datasets generated and/or analyzed during the current study are available from the corresponding author on reasonable request.
