# Supplementary material for: Immunogold labeling of synaptic vesicle proteins in developing hippocampal neurons
Source: Mol Brain. 2020 Jan 20;13:9. doi: 10.1186/s13041-020-0549-x (PMC6971973; doi:10.1186/s13041-020-0549-x)
Supplement: Supplementary file 1 — Additional file 1. Optimal fixation conditions for each antibody. [file 13041_2020_549_MOESM1_ESM.pdf]

Additional File 1. OPTIMAL FIXATION CONDITIONS FOR EACH ANTIBODY

Various fixation protocols were tested to determine the optimal fixation condition for each antibody to achieve significant labeling and structural preservation for interpretation at the EM level. Parallel controls were carried out for different fixation conditions. In all cases, no specific labeling was observed in control samples where primary antibody was omitted.

1. **synaptophysin** (rabbit pAb, Dako) – optimally fixed with 4% paraformaldehyde and 0.05% glutaraldehyde in PBS for 45 min. It also gave good signals after fixation with 4% paraformaldehyde in PBS for 60 min.
2. **SV2** (mouse mAb, clone 10H3, a gift from Dr. E. S. Schweitzer) – optimally fixed with 4% paraformaldehyde and 0.05% glutaraldehyde in PBS for 60 min. It also gave good signals after fixation with (1) 4% paraformaldehyde in PBS for 60 min, (2) 4% paraformaldehyde and 0.1% glutaraldehyde in PBS for 30 min and (3) 2% acrolein in PBS for 1 min, followed by 4% paraformaldehyde in PBS for 30-60 min.
3. **VAMP** (synaptobrevin, mouse mAb, clone SP10, Chemicon) - optimally fixed with 4% paraformaldehyde in PBS for 45 min. Longer fixation yielded lower labeling. This is an IgM antibody, so an IgG secondary antibody was used instead of the usual Fab' secondary antibody..
4. **synaptotagmin** (p65, mouse mAb, clone ASV30, Stressgen) - optimally fixed with 4% paraformaldehyde in PBS for 60 min. This antibody did not yield acceptable labeling after fixation with (1) 4% paraformaldehyde and 0.02% glutaraldehyde in PBS for 30 min, and (2) 2% acrolein in PBS for 1 min, followed by 4% paraformaldehyde in PBS for 30 min.
5. **synapsin I** (mouse mAb, clone 46.1, Synaptic Systems) - optimally fixed with 4% paraformaldehyde in PBS for 60 min. This antibody did not yield acceptable labeling after fixation with 4% paraformaldehyde and 0.02% glutaraldehyde in PBS for 30 min.
6. **synuclein** (mouse mAb, clone 42, BD Biosciences) - optimally fixed with 4% paraformaldehyde in PBS for 60 min.
7. **SANP-25** (mouse mAb, clone SP14, Chemicon) - optimally fixed with 4% paraformaldehyde and 0.02% glutaraldehyde in PBS for 45 min. It also gave good signals after fixation with 4% paraformaldehyde in PBS for 60 min.
8. **Bassoon** (mouse mAb, clone SAP7F407, Stressgen) – optimally fixed with 4% paraformaldehyde in PBS for 60 min. This antibody did not yield acceptable labeling after fixation with (1) 4% paraformaldehyde and 0.02% glutaraldehyde in PBS for 30 min, and (2) 2% acrolein in PBS for 1 min, followed by 4% paraformaldehyde in PBS for 30 min.
9. **Piccolo** (Guinea pig pAb, a gift from Dr. Eckart Gundelfinger) - optimally fixed with 4% paraformaldehyde and 0.05% glutaraldehyde in PBS for 45 min. It also yielded good signals after fixation with (1) 4% paraformaldehyde in PBS for 60 min and (2) 2% acrolein in PBS for 1 min, followed by 4% paraformaldehyde in PBS for 30-60 min.
