# Supplementary material for: Immunogold labeling of synaptic vesicle proteins in developing hippocampal neurons
Source: Mol Brain. 2020 Jan 20;13:9. doi: 10.1186/s13041-020-0549-x (PMC6971973; doi:10.1186/s13041-020-0549-x)
Supplement: Supplementary file 2 — Additional file 2. Immunogold labeling of SV proteins in young axons. [file 13041_2020_549_MOESM2_ESM.pdf]

Additional File 2. Immunogold labeling of SV proteins in young axons

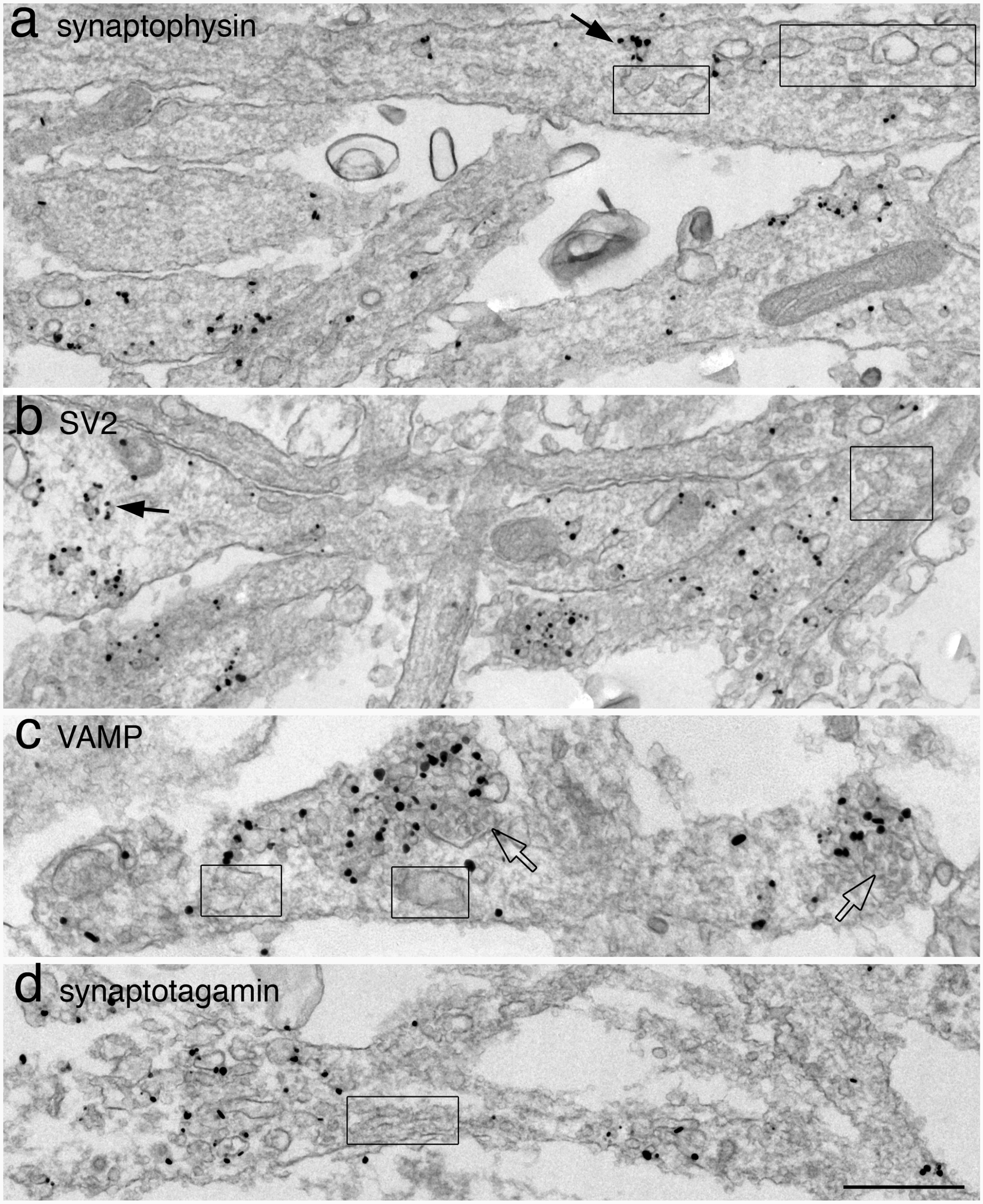

Labels for these four SV integral membrane proteins are specifically localized on membranous sturctures. However, not all vesicles/vacuoles are labeled for each of these antibodies (boxed areas show unlabeled vesicles). Samples are from dissociated hippocampal cultures at 3 days in vitro. Open arrows points to multivesicular bodies. Scale bar = 500 nm.
