# Supplementary material for: Immunogold labeling of synaptic vesicle proteins in developing hippocampal neurons
Source: Mol Brain. 2020 Jan 20;13:9. doi: 10.1186/s13041-020-0549-x (PMC6971973; doi:10.1186/s13041-020-0549-x)
Supplement: Supplementary file 3 — Additional file 3. Comparison among SV membrane protein transport aggregate, AZ protein transport aggregate, and cluster of SV-like vesicles. [file 13041_2020_549_MOESM3_ESM.pdf]

Additional File 3. Comparison among SV membrane protein transport aggregate, AZ protein transport aggregate, and clusters of SV-like vesicles.

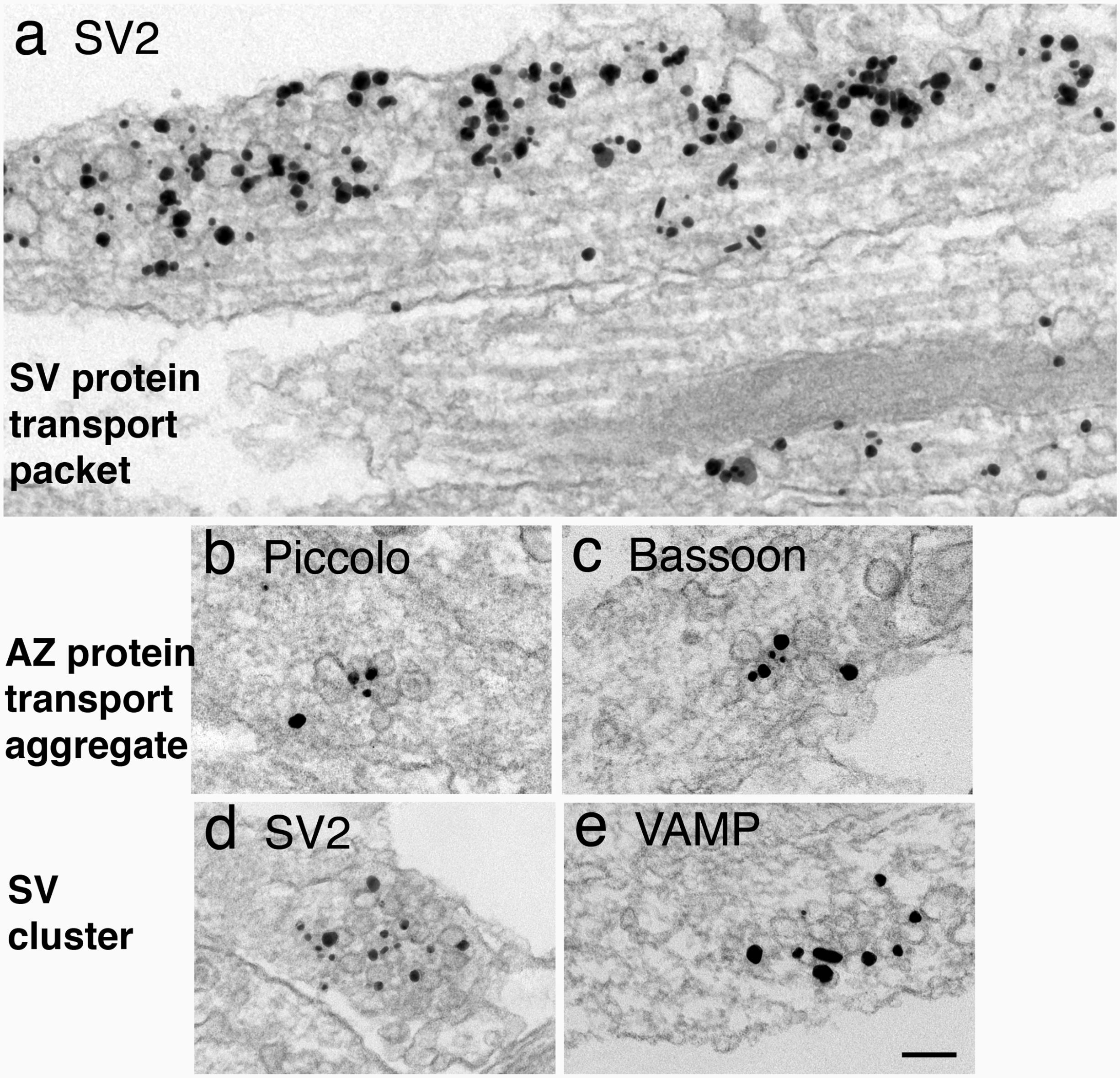

SV protein transport packets are variable in size, with some greater than 2 μm in length (SV2-labeled aggregate on top in a) and others smaller with fewer vesicles (labeled aggregate on bottom in a). On the other hand, AZ transport aggregates are much smaller at ~0.2 μm (b & c). Finally, d & e show clusters of uniform-sized SVs labeled for SV membrane proteins. Samples are from dissociated hippocampal cultures at 3 (c, d), 4 (a, e), 5 (b) days in vitro. Scale bar = 100 nm.
