# Supplementary material for: Immunogold labeling of synaptic vesicle proteins in developing hippocampal neurons
Source: Mol Brain. 2020 Jan 20;13:9. doi: 10.1186/s13041-020-0549-x (PMC6971973; doi:10.1186/s13041-020-0549-x)
Supplement: Supplementary file 4 — Additional file 4. Serial sections through a young axon at 5 days in culture labeled with SV2 antibody. [file 13041_2020_549_MOESM4_ESM.pdf]

Additional File 4. Serial sections through a young axon at 5 days in culture labeled with SV2 antibody.

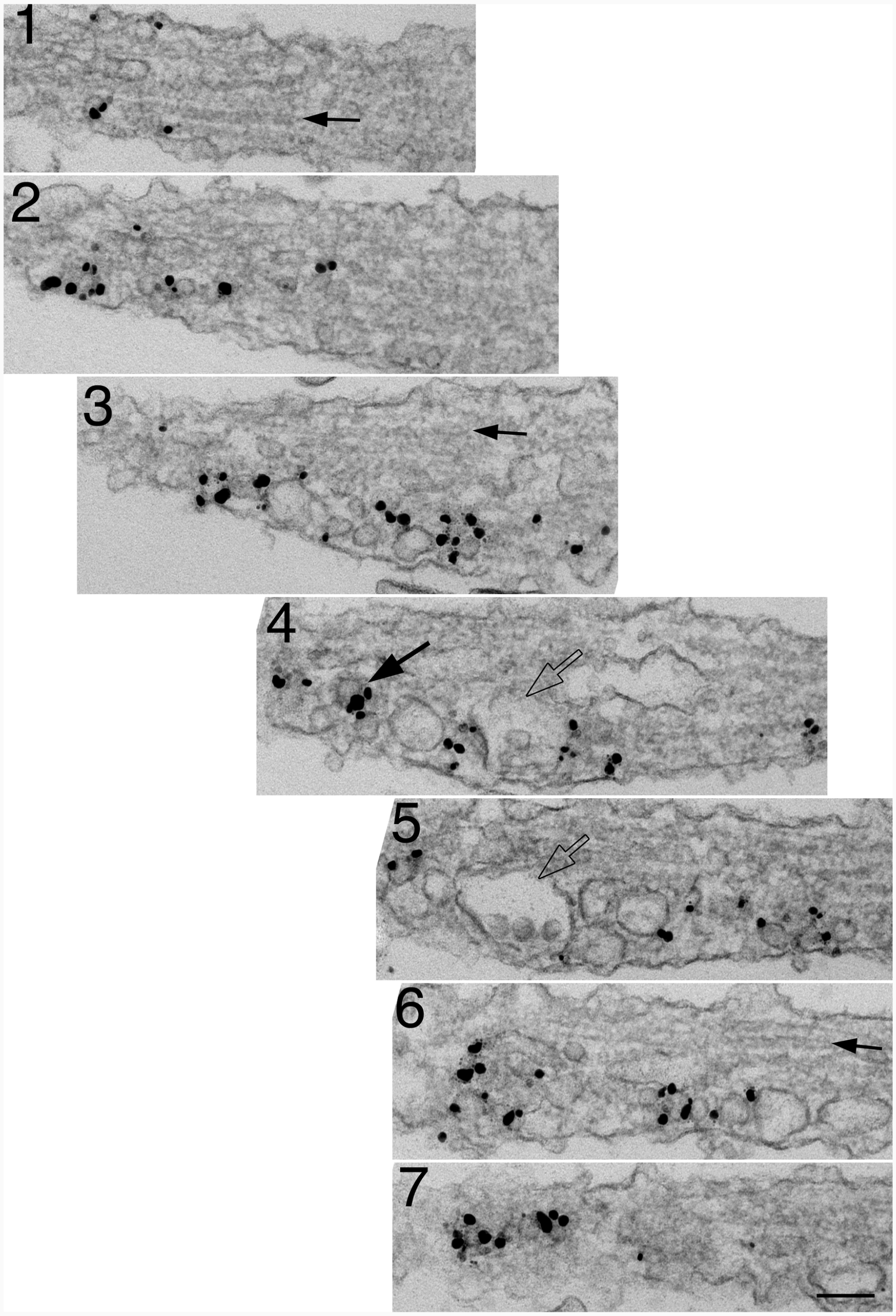

Label for SV2 is specifically localized on membranous structures of various sizes. A large arrow in section #4 points to a labeled coated vesicle. Open arrows in #4 & 5 point to a multivesicular body. Sample was fixed with acrolein initially to preserve microtubules (small arrows in sections #1, 3, 6) Scale bar = 100 nm.
